# Insights into a Novel *bla*_KPC-2_ **-**Encoding IncP-6 Plasmid Reveal Carbapenem-Resistance Circulation in Several *Enterobacteriaceae* Species from Wastewater and a Hospital Source in Spain

**DOI:** 10.3389/fmicb.2017.01143

**Published:** 2017-06-28

**Authors:** Yancheng Yao, Fernando Lazaro-Perona, Linda Falgenhauer, Aránzazu Valverde, Can Imirzalioglu, Lucas Dominguez, Rafael Cantón, Jesús Mingorance, Trinad Chakraborty

**Affiliations:** ^1^Institute of Medical Microbiology, Justus Liebig University Giessen and German Center for Infection Research, Partner Site Giessen-Marburg-LangenGiessen, Germany; ^2^Servicio de Microbiología, Hospital Universitario La Paz and Instituto de Investigación Sanitaria Hospital La PazMadrid, Spain; ^3^Centro de Vigilancia Sanitaria Veterinaria (VISAVET), Universidad Complutense de MadridMadrid, Spain; ^4^Red Española de Investigación en Patología Infecciosa SpainMadrid, Spain; ^5^Servicio de Microbiología, Hospital Universitario Ramón y Cajal and Instituto Ramón y Cajal de Investigación SanitariaMadrid, Spain

**Keywords:** KPC2 carbapenemase, IncP-6 plasmid, *Enterobacteriaceae*, wastewater and hospital source, Spain

## Abstract

Untreated wastewater, particularly from hospitals and other healthcare facilities, is considered to be a reservoir for multidrug-resistant bacteria. However, its role in the spread of antibiotic resistances in the human population remains poorly investigated. We used whole genome sequencing to analyze 25 KPC-2-producing *Enterobacteriaceae* isolates from sewage water collected during a 3-year period and three clinical *Citrobacter freundii* isolates from a tertiary hospital in the same collection area in Spain. We detected a common, recently described, IncP-6 plasmid carrying the gene *bla*_KPC-2_ in 21 isolates from both sources. The plasmid was present in diverse environmental bacterial species of opportunistic pathogens such as *C. freundii, Enterobacter cloacae, Klebsiella oxytoca*, and *Raoultella ornithinolytica*. The 40,186 bp IncP-6 plasmid encoded 52 coding sequences and was composed of three uniquely combined regions that were derived from other plasmids recently reported in different countries of South America. The region harboring the carbapenem resistance gene (14 kb) contained a Tn*3* transposon disrupted by an IS*Apu*-flanked element and the core sequence composed by IS*Kpn6*/*bla*_KPC-2_/Δ*bla*_TEM-1_/IS*Kpn27*. We document here the presence of a novel promiscuous *bla*_KPC-2_ plasmid circulating in environmental bacteria in wastewater and human populations.

## Introduction

Antibiotic resistance is currently one of the biggest threats to global health. The emergence and spread of carbapenemase- producing *Enterobacteriaceae* (CPE) has become a major problem for clinical and public health since they are resistant to all β-lactam antibiotics and also frequently to other antibiotic classes, limiting the treatment options. CPE are associated with a wide spectrum of opportunistic infections, including bloodstream, urinary and respiratory tract infections and CPE nosocomial outbreaks have been globally reported (Nordmann et al., 2012a; Munoz-Price et al., 2013).

Carbapenemase producing *Enterobacteriaceae* harboring the *bla*_KPC-2_ gene are among the most frequently isolated. Like other carbapenemases (Cuzon et al., 2010), *bla*_KPC-2_ is usually carried on mobile genetic elements such as plasmids and transposons (Shen et al., 2009; Cuzon et al., 2011) and thus, can spread horizontally to naive bacteria contributing to an increase in the reservoir of antibiotic resistance in both environmental and clinical *Enterobacteriaceae*. The *bla*_KPC-2_ gene has been found on several plasmids with different incompatibility (Inc) groups, usually IncFII, IncL/M, IncN, or IncA/C (Ruiz-Garbajosa et al., 2013; Chen et al., 2014; Chmelnitsky et al., 2014; Yao et al., 2014), and only rarely on other plasmids like IncP (Dai et al., 2016) and IncX (Ho et al., 2013). The best known genetic environment of *bla*_KPC-2_ is the Tn*4401* transposon (Chmelnitsky et al., 2014). However, other structures based on Tn*3* transposons have been also reported from China (Shen et al., 2009) as well as from Argentina (Gomez et al., 2011), Brazil (Andrade et al., 2011), and Colombia (Naas et al., 2013). Recently, Dai et al. (2016) have reported the first detection of an IncP-6 plasmid encoding a *bla*_KPC-2_ in a clinical *Pseudomonas aeruginosa* isolate from a Chinese public hospital.

In this work, we analyze by whole genome sequencing (WGS) 25 bacterial isolates of different enterobacterial species from untreated urban effluents of the northern area of Madrid (Spain), sampled between 2011 and 2013, as well as three clinical *Citrobacter freundii* isolates that caused a fatal outbreak in a tertiary hospital (Gómez-Gil et al., 2010) in the same catchment area. We detected in isolates from both sources an IncP-6 plasmid harboring a *bla*_KPC-2_ gene (p121SC21) similar to the one described by Dai et al. (2016). We describe the genetic structure of the plasmid, in particular the genetic environment surrounding the *bla*_KPC-2_ gene and give an overview of plasmid diversity and the broad host range of this new plasmid.

## Materials and Methods

### Sampling and Bacterial Identification

Water samples were obtained from untreated urban effluents (including domestic and hospital effluents from an area of nearly 5 × 10^5^ inhabitants) in July 2011, March 2012, and March 2013 in Madrid (Spain). Samples were filtered and clarified through a Millipore vacuum system (STERIFIL). To obtain carbapenem resistant isolates 100 μl of filtered samples were plated as follows: a 10^-2^ dilution onto MacConkey agar supplemented with 2 mg/L of cefotaxime and 2 mg/L of ceftazidime for the samples in 2011 and undiluted sample onto Supercarba medium (Nordmann et al., 2012b) for the samples after 2011. Plates were incubated overnight at 37°C and one isolate per morphotype (colony morphology) was selected for further analysis. Bacterial identification was performed using mass spectrometry MALDI-TOF MS (Bruker Daltonics GmbH; Bremen, Germany). Pulsed field gel electrophoresis (PFGE) was performed using *XbaI*-digested genomic DNA separated in a CHEF-DRIII system (Bio-Rad, La Jolla, CA, United States). One isolate per pulsotype was selected for further analysis. The presence of genes encoding carbapenemases was detected by PCR assay. Conjugation experiments were performed for the isolates from 2011 and 2012 using the recipient strain *Escherichia coli* BM21 (Nalidixic and Rifampin resistant) and transconjugants were obtained (Supplementary Tables).

### Bacterial Genome Sequencing and Analyses

Twenty-five KPC-2-producers from the wastewater and three *C. freundii* KPC-2-producers from an outbreak in La Paz Hospital [Madrid; from October 2009 to January 2010, (Gómez-Gil et al., 2010)] were sequenced. Whole genome DNA was isolated from an overnight culture using the Purelink^®^ Genome DNA Mini kit (Thermo Fischer Scientific, Dreieich, Germany).

DNA sequencing libraries were prepared using Nextera XT kit (Illumina, San Diego, CA, United States) according to the manufacturer’s instructions and sequenced using the Illumina MiSeq^®^ system (Illumina, San Diego, CA, United States) with 2 × 300 base paired-end sequencing. Reads were assembled using CLC Genomics Workbench version 7.5.1 (CLC QIAGEN Bioinformatics, Denmark).

Antibiotic resistance genes were identified with ResFinder^1^. Plasmid replicons and plasmid incompatibility groups were predicted using PlasmidFinder^2^. To assemble the plasmids harboring *bla*_KPC-2_, multi-genome alignments and contig ordering were performed using MAUVE (Darling et al., 2010) and gap closure was done with SeqManPro (DNASTAR Program package). ORF finding and annotation was preliminary performed with RAST^3^ and finally manually corrected using BLAST.

Details of sampling, results of conjugation experiments, genome sequencing and sequence primary analyses to determine MLST (*in silico*), plasmid incompatibility groups and antibiotic resistance genes are given in the Supplementary Tables. All the sequencing data is deposited under the project accession number PRJEB19777.

## Results

### Selection of Wastewater KPC-Producing Isolates for Sequencing

Forty-three KPC-2-producing *Enterobacteriaceae* were isolated from the wastewater samples in three different screenings (6 were isolated on MacConkey-plates in 2011, 28 and 9 were isolated on Supercarba-plates in 2012 and 2013, respectively). Twenty-five isolates were selected for WGS taking at least one representative of each species and one isolate per pulsotype. (*C. freundii, n* = 5; *C. braakii, n* = 1; *C. farmeri, n* = 1; *Enterobacter cloacae/asburiae, n* = 5; *Klebsiella oxytoca, n* = 6; *K. pneumoniae, n* = 1; *Kluyvera ascorbata, n* = 1; *Kluyvera cryocrescens, n* = 2, and *Raoultella ornithinolytica, n* = 3).

### IncP-6 Plasmids Carrying *bla*_KPC-2_, the Plasmid p121SC21-KPC2 and Its Derivatives in Wastewater Isolates

Twenty-four out of the twenty-five sequenced wastewater isolates (apart from an isolate *K. oxytoca*) harbored the *bla*_KPC-2_ gene. In 19 of them, the *bla*_KPC-2_ gene was localized on an IncP-6 plasmid, whereas in the other five isolates the plasmids carrying *bla*_KPC-2_ belonged to the incompatibility groups IncX3, IncN, and IncU.

**FIGURE 1:**
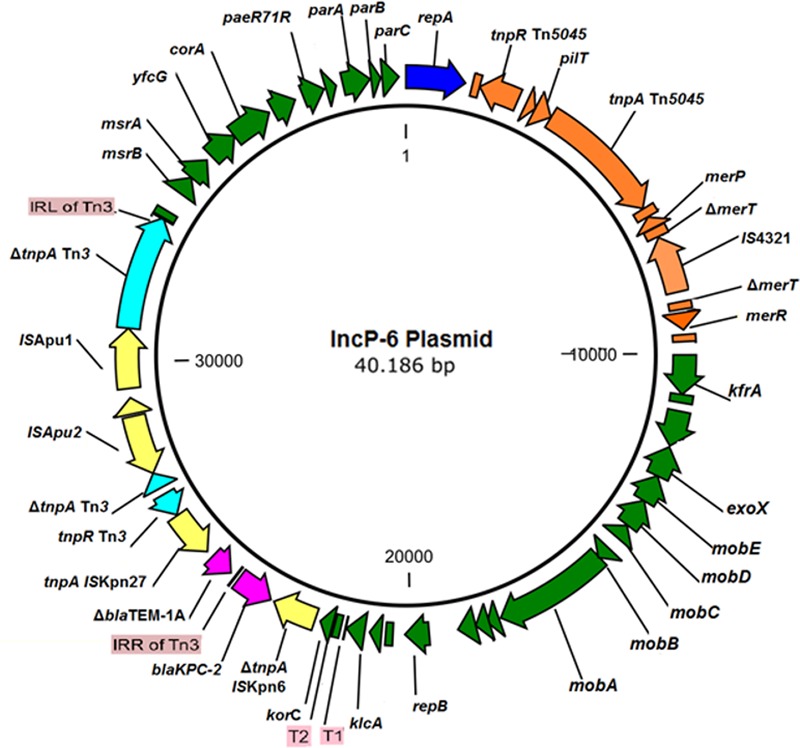
Genetic Map of the p121SC21-KPC2. The backbone of the plasmid contains one region responsible for mobilization and another for replication and maintenance (both indicated in dark green). The gene *bla*_KPC-2_ (fucsia arrow) is located upstream of an interrupted *bla*_TEM-1_ gene and flanked by IS*Kpn6* and IS*Kpn27*. It is adjacent to a Tn*3* transposon with an IS*Apu*-mediated interrupted transposase.

**FIGURE 2:**
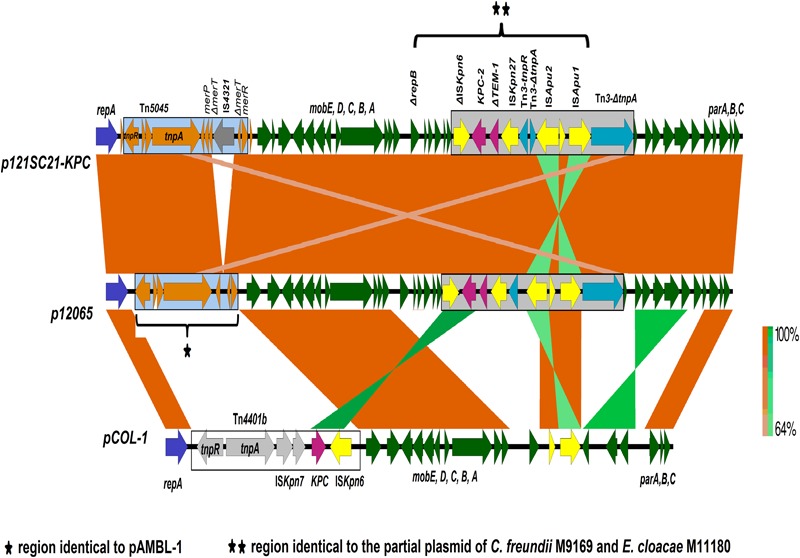
Structure comparison of the p121SC21-KPC2 with p12065 and pCOL-1. Genes (open reading frames) and their corresponding transcriptional orientations are indicated by broad horizontal arrows. The orange- and green-shaded areas show identical regions among the structures compared, in the same orientation or inverted, respectively.

The IncP-6 plasmid from the wastewater isolate *C. freundii* 121SC21 obtained in 2012, designated as p121SC21-KPC2 could be closed and analyzed. The plasmid p121SC21-KPC2 is 40,186 bp in size with an average G/C-content of 58%. It comprises 52 open reading frames (ORF), of which 37 encode proteins with known functions and 15 hypothetical proteins (**Figure 1**). The plasmid is composed of four different regions. The backbone of the plasmid contains a region responsible for mobilization (8.1 kb) and another for replication and maintenance (7.4 kb) and also encodes an anti-oxidative system *mrsA*/*mrsB* together with a glutathione *S*-transferase (**Figure 1**). These two regions are highly similar to the plasmid pCOL-1 (accession number NC022346.1) from the *P. aeruginosa* strain ST308 (Naas et al., 2013) with a nucleotide identity of > 98% (**Figure 2**). Within the backbone, p121SC21-KPC2 harbored an inserted region containing a Tn*5045*-associated mercury resistance operon, which was similar to that of pAMBL-1 (KP873172.1) with exception of a disrupted *merT* gene due to the insertion of an IS*4321* insertion sequence (**Figure 2**). Instead of a Tn*4011* in pCOL-1, the *bla*_KPC-2_ gene was located in another Tn*3*-based transposon (14.1 kb) with a disrupted Tn*3* transposase that matched 100% to a partial sequence (10.0 kb) of the clinical isolates *C. freundii* M9169 and *E. cloacae* M11180 from Argentina (Gomez et al., 2011). The genetic arrangement was: IS*Kpn6* + *bla*_KPC-2_ + Δ*bla*_TEM-1_ + IS*Kpn27* + Tn*3*-*tnpR* + Tn*3*-*ΔtnpA* + IS*Apu2* + orf + IS*Apu1* + Tn*3*-*ΔtnpA* (**Figure 2**). The p121SC21-KPC2 was almost identical (99% similarity) to the plasmid p10265-KPC (GenBank accession number KU578314) that was isolated from a Chinese public hospital in 2010. The differences were the presence of an IS*4321-tnpA* disrupting the *merT* gene and some minor changes surrounding the *repA* and *klcA* genes.

**FIGURE 3:**
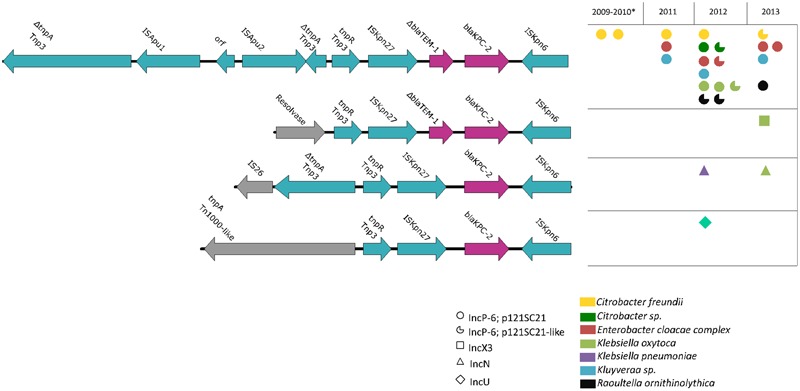
Genetic *bla*_KPC-2_ environments and their distributions. Four different genetic environments of the gene *bla*_KPC-2_ were present in plasmids of different incompatibility groups of IncP-6, IncX3, IncN, and IncU. The locus adjacent to the Tn*3*-*tnpR* was variable. The predominant genetic environment, IncP-6 type, contained a disrupted Tn*3* transposase by insertion of an IS*Apu1*/IS*Apu2*-element and was encountered in 21 isolates belonging to several species during the whole period and in isolates of both clinical and wastewater sources.

Combining contig sequences with read mapping, we detected identical IncP-6 plasmids in a total of 13 wastewater isolates of different species (**Figure 3**). This included the species *C. farmeri* (*n* = 1), *C. freundii* (n = 3), *E. cloacae complex* (including *E. asburiae*, n = 4), *K. oxytoca* (*n* = 2), *K. cryocrescens* (*n* = 2), and *R. ornithinolytica* (*n* = 1), suggesting a broad host range plasmid and its persistence in the wastewater. The remaining six isolates harbored IncP-6 plasmids whose sequences were not complete (**Figure 3**, notched circles indicated). Two of them differed from p121SC21-KPC2 by the absence of about 100 bp sequence at the ends of the sequences, probably due to low sequencing coverage and are regarded as the same plasmid. Three p121SC21-KPC2-like plasmids with larger differences were identified in the other four isolates. They had a common region of 30 kb, between the Tn*5045* and *repB*, identical to that of p121SC21-KPC2 and several major variations localized in the region of mobilization and consisting of different mobile genetic elements (Supplementary Figure 1).

### Presence of Identical IncP-6 Plasmid in the Hospital Outbreak Isolates

Among the three clinical *C. freundii* isolates from a tertiary a hospital in the same area collected from October 2009 to January 2010 we found a plasmid with 100% identity to p121SC21-KPC2 in isolate HULP-6197, and an almost identical plasmid with a 185 bp deletion in the *merR* gene in *C. freundii* HULP-4836 (**Figure 3**). Phylogenetic analysis and *in silico* MLST revealed that the clinical *C. freundii* isolates were clonally related. On the contrary, *C. freundii* from wastewater were diverse and were neither clonally related between themselves nor with the isolates from the hospital.

### Other Plasmids Carrying *bla*_KPC-2_ in Wastewater Isolates

In addition to the 21 isolates from both clinical and wastewater sources that carried the p121SC21-KPC2 or a p121SC21-KPC2-like plasmid, we identified five wastewater isolates harboring *bla*_KPC-2_-carrying plasmids of different incompatibility groups, including IncN (*n* = 2), IncX3 (*n* = 1), IncU (*n* = 1), and a non-typeable plasmid (*n* = 1). In addition to being on different plasmid types these were variable with respect to the genetic arrangements surrounding the *bla*_KPC-2_ gene (**Figure 3**). The variant IncX3 presented a structure similar to pKP13d (accession number CP003997) (Ramos et al., 2014) and contained a putative resolvase instead of the disrupted Tn*3* transposase and was interrupted by an insertion of IS*Apu1*/IS*Apu2* in the IncP-6 plasmid (**Figure 3**). In the IncN plasmid the Tn*3* transposase was disrupted by an IS*26*. In the IncU plasmid, this locus was replaced by a partial Tn*1000*-like transposon (**Figure 3**). Furthermore, the Δ*bla*_TEM-1_ sequence downstream of IS*Kpn27* was absent in the plasmids of type IncN and IncU (**Figure 3**).

## Discussion

In this work, we have analyzed the genomes of 28 carbapenem- resistant isolates from a wastewater plant and a nosocomial outbreak in a tertiary hospital in the same catchment area in Spain and ascertained the genetic environments of *bla*_KPC-2_ comprehensively. In total, 2/3 clinical and 24/25 wastewater sequenced isolates harbored a *bla*_KPC-2_ gene. The *bla*_KPC-2_ gene was located on different plasmids of the incompatibility group IncP-6 (*n* = 21), IncN (*n* = 2), IncX3 (*n* = 1) or IncU (*n* = 1) as well as a non-typeable group (*n* = 1). The non-recovery of *bla*_KPC-2_ in the genome of two isolates might be attributed to the loss of their KPC-2 plasmids during storage.

The *bla*_KPC-2_-encoding IncP-6 plasmid in this study was experimentally confirmed to be transferable by conjugation and carried genes for stabilization and conjugation. The complete IncP-6 plasmid sequence, present predominately in 17 different isolates, carried the *mob*C, *mob*D, and *mob*E genes that are highly similar, with a nucleotide identity > 95%, to those on pTC-FC2 described in Guynet et al. (2011). The *mob*CDE operon adjacent to the conjugative transfer origin (*ori*T) was syntenic to the *stb*ABC operon that has a role on plasmid conjugation (Guynet et al., 2011).

Although IncP-6 plasmids are naturally isolated from *P. aeruginosa* (Boronin, 1992) the predominance of IncP-6 plasmids carrying *bla*_KPC-2_ in diverse species from both clinical and wastewater sources suggest that they have a broad host range and are associated with bacteria that have the ability to persist in the environment for long periods.

In other studies, the predominant plasmid types harboring *bla*_KPC-2_ gene were either of IncA/C incompatibility group or non-typeable from isolates obtained from a Chinese wastewater treatment plant (Zhang et al., 2012) whereas an IncN plasmid was predominant in isolates obtained from wastewater in Germany (Eikmeyer et al., 2012). It appears that different plasmid types can predominate in the wastewater of geographically distant catchment areas and that the IncP6 plasmid was unique to the niche in our study.

The IncP-6 plasmid was present in eight different *Enterobacteriaceae* species, mainly in *Citrobacter* sp. (*C. freundii, n* = 5), *Enterobacter* sp. (*E. asburiae, n* = *4*), *Klebsiella* sp. (*K. oxytoca, n* = 3), *Raoultella* sp. (*R. ornithinolytica, n* = 3), and *Kluyvera* sp. (*K. cryocrescens, n* = 2) (Supplementary Figure 2). Genome-based phylogenetic analyses indicated a diverse and non-clonal relationship between isolates within each species, whereas the same *bla*_KPC-2_ encoding IncP-6 plasmid was present in different isolates and might play an important role in the horizontal dissemination of KPC-2 carbapenem resistance in the wastewater and the spread from the wastewater to human and vice versa.

The composite structure of the IncP-6 plasmid and the highest similarity with pCOL-1 of *P. aeruginosa* strain ST308 from Colombia (Naas et al., 2013) for the backbone region sequence and with pAMBL-1 of *P. aeruginosa* strain PAO1 from Brazil for the sequence of a mercury resistance cassette suggest an environmental origin. Remarkably, the *bla*_KPC-2_ region is identical to the plasmid sequences of clinical *C. freundii* M9169 and *Enterobacter cloacae* M11180 from Argentina (Gomez et al., 2011). This association of different segments of the IncP-6 plasmid to environmental opportunistic pathogens from South America suggests a geographic origin on that continent prior to its introduction into Spain.

Most studies to date on the occurrence of *bla*_KPC-2_ indicate its association with the Tn*4401*-based transposon and consider it a major element responsible for the worldwide spread of this resistance gene. However, the Tn*4401-*associated *bla*_KPC-2_ was more frequently reported in isolates from hospital rather than from urban wastewaters. In all of the known Tn*4401* isoforms (a, b, c, to g), the gene *bla*_KPC-2_ was flanked by insertion sequences like IS*Kpn7* and IS*Kpn6*. This is also the case with IncP-6 plasmid p121SC21-KPC2. However, they are also commonly associated with a juxtaposed Tn*3* transposon with a disrupted Tn*3*-transposase as the result of the insertion of an unknown ORF flanked by IS*Apu* elements. Apart from minor variations in the mobilization region, the IncP-6 plasmid is highly conserved. Remarkably, in all of the detected plasmids carrying *bla*_KPC-2_, including IncP-6, IncX3, IncN, and IncU, the gene *bla*_KPC-2_ was always detected as a unit, flanked by the insertion elements IS*Kpn27* and IS*Kpn6*.

Our work adds to the general notion that antibiotic resistance genes have no boundaries. Plasmids carrying these genes can easily spread worldwide while urban wastewater may act as reservoir and reactor of antimicrobial resistance within bacterial populations. Moreover, wastewater bacterial communities create new opportunities for recombination and rearrangements, leading to the appearance of new plasmids that ultimately could reach the human population and vice versa. These results also underline the need to seek for strategies that directly limit clonal plasmid spread into multitudes of bacterial species.

The emergence of CPE limits therapeutic options, particularly when such bacteria become also resistant to colistin (Falgenhauer et al., 2016). Thus, effective surveillance and interruption of the spread of CPE is urgently needed. Direct detection of *bla*_KPC-2_ plasmid of carbapenem resistant isolates in wastewater may be used as an effective epidemiological indicator. The surveillance of antimicrobial resistance genes in hospital sewage and urban effluents could provide complementary information to the monitoring data of clinical samples and can be useful to generate a comprehensive picture of sources and distribution of antimicrobial resistance in the environment.

## Author Contributions

TC, YY, JM, and RC designed the study; FL-P, AV, CI, LD, and RC performed experiments; YY, FL-P, LF, CI, and TC analyzed data. YY, LF, AV, CI, LD, RC, JM, and TC contributed reagents, materials and analysis tools. YY, FL-P, LF, and TC wrote this manuscript.

## Conflict of Interest Statement

The authors declare that the research was conducted in the absence of any commercial or financial relationships that could be construed as a potential conflict of interest.

## Footnotes

1 http://cge.cbs.dtu.dk/services/ResFinder/

2 https://cge.cbs.dtu.dk//services/PlasmidFinder/

3 http://rast.nmpdr.org/
