# Supplementary material for: Insights into a Novel blaKPC-2 -Encoding IncP-6 Plasmid Reveal Carbapenem-Resistance Circulation in Several Enterobacteriaceae Species from Wastewater and a Hospital Source in Spain
Source: Front Microbiol. 2017 Jun 28;8:1143. doi: 10.3389/fmicb.2017.01143 (PMC5487458; doi:10.3389/fmicb.2017.01143)
Supplement: Supplementary file 1 [file Data_Sheet_1.pdf]

frontiers

Supplementary Material

Insights into the circulation of carbapenem resistance in *Enterobacteriaceae* from wastewater and a hospital source in Spain by a novel *bla*$_{KPC-2}$-encoding IncP-6 plasmid

Yancheng Yao [1,†], Fernando Lazaro-Perona [2,†], Linda Falgenhauer[1], Aránzazu Valverde, [4,5], Can Imirzalioglu[1], Lucas Dominguez[4], Rafael Cantón [3,5], Jesús Mingorance [2,5], Trinad Chakraborty [1,*]

[1] Institute of Medical Microbiology, Justus Liebig University Giessen, and German Center for Infection Research (DZIF), Partner Site Giessen-Marburg-Langen, Campus Giessen, Giessen, Germany

[2] Servicio de Microbiología. Hospital Universitario La Paz and Instituto de Investigaciones Sanitarias Hospital La Paz (IdiPAZ). Madrid, Spain

[3] Servicio de Microbiología. Hospital Universitario Ramón y Cajal and Instituto Ramón y Cajal de Investigaciones Sanitarias (IRYCIS). Madrid, Spain

[4] Centro de Vigilancia Sanitaria Veterinaria (VISAVET). Universidad Complutense, Madrid, Spain

[5] Red Española de Investigación en Patología Infecciosa Spain (REIPI), Madrid, Spain

[†] These authors contributed equally to the manuscript.

*** Correspondence:**
Trinad Chakraborty
Trinad.chakraborty@mikrobio.med.uni-giessen.de

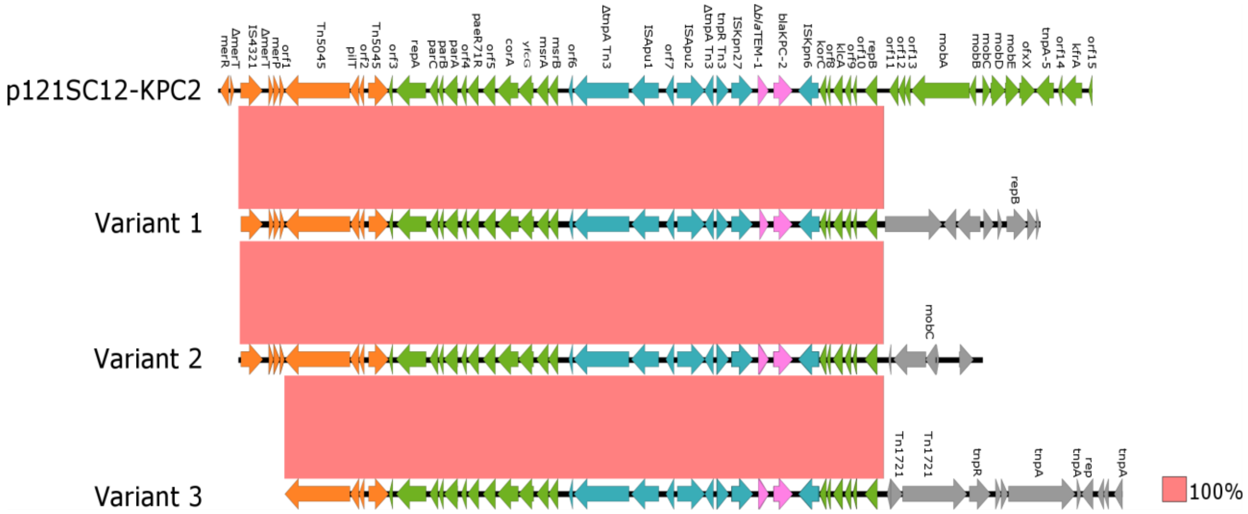

Supplementary Figure 1: Diversity of IncP-6 KPC2-Plasmids in Wastewater. Three different incomplete IncP-6 plasmids presented a 30kb common region with the p121SC21-KPC2 and the major variations related with insertions in the region of mobilization upstream of *repB* gene, suggest an active process of recombination. Variant 1 present in isolate 121SC10 and 121SC39, variant 2 in 121SC57 and variant 3 in 121SC85.

	KPC-2 positive/ total sequenced	IncP-6 closed	IncP-6 derivates	IncN	IncU	IncX3	other
Citrobacter freundii	5/ 5	● (blue) ● (orange) ● (green)		○ (orange)	○ (orange)		
Citrobacter braakii	1/ 1		○ (orange)				
Citrobacter farmeri	1/ 1	● (orange)					
Enterobacter cloacae	4/ 4	● (blue) ● (orange) ● (green) ● (green)					
Enterobacter asburiae	1/ 1						○ (orange)
Klebsiella oxytoca	5/ 6	● (orange) ● (orange)	○ (orange)	○ (green)		● (green)	
Klebsiella pneumoniae	1/ 1			○ (orange)			
Kluyvera ascorbata	1/ 1	● (green)					
Kluyvera cryocrescens	2/ 2	● (blue) ● (orange)					
Raoultella ornithinolythica	3/ 3	● (green)	○ (orange) ○ (orange)				
	24/25						

Solid- and hollow- circle indicate complete and gaped plasmid sequence, respectively.

● (blue) isolates from 2011 ● (orange) isolates from 2012 ● (green) isolates from 2013

Supplementary Figure 2: Detected KPC2-Plasmids in Wastewater. We determined the bla_{KPC-2} gene in 24 of 25 sequenced wastewater isolates. The KPC-2 was predominantly present in an IncP-6 plasmid distributed in eight *Enterobacteriaceae* species.

Supplementary Table 1: KPC-2 isolates (n=46)

KPC-2 positive isolates obtained from the urban effluent between 2011 and 2013 and from an outbreak at a hospital in Madrid 2009-2010								
**ISOLATE**	**Year**	**Microorganism**	**Carbapenemase**	**Plasmid size based on s1-hybridization (kb)**	**Transconjugant Size (kb) ***	**Source**	**Institution**	**sequenced**
121SC10	2012	*Raoultella ornithinolythica*	KPC-2	50	50	urban effluent	VISAVET	1
121SC13	2012	*Citrobacter farmeri*	KPC-2	ND	NO TC	urban effluent	VISAVET	1
121SC2	2012	*Enterobacter asburiae*	KPC-2	ND	NO TC	urban effluent	VISAVET	
121SC21	2012	*Citrobacter freundii*	KPC-2	50	NO TC	urban effluent	VISAVET	1
121SC25	2012	*Citrobacter freundii*	KPC-2	ND	ND	urban effluent	VISAVET	
121SC29	2012	*Raoultella ornithinolythica*	KPC-2	ND	ND	urban effluent	VISAVET	
121SC3	2012	*Enterobacter asburiae*	KPC-2	ND	ND	urban effluent	VISAVET	1
121SC32	2012	*Enterobacter asburiae*	KPC-2	50	NO TC	urban effluent	VISAVET	1
121SC33	2012	*Citrobacter freundii*	KPC-2	ND	NO TC	urban effluent	VISAVET	
121SC37	2012	*Citrobacter freundii*	KPC-2	50	NO TC	urban effluent	VISAVET	
121SC39	2012	*Raoultella ornithinolythica*	KPC-2	50	50	urban effluent	VISAVET	1
121SC5	2012	*Raoultella ornithinolythica*	KPC-2	60	NO TC	urban effluent	VISAVET	
121SC53	2012	*Enterobacter asburiae*	KPC-2	ND	NO TC	urban effluent	VISAVET	
121SC54	2012	*Citrobacter freundii*	KPC-2	50	50	urban effluent	VISAVET	1
121SC57	2012	*Citrobacter braakii*	KPC-2	ND	NO TC	urban effluent	VISAVET	1
121SC58	2012	*Citrobacter freundii*	KPC-2	50	NO TC	urban effluent	VISAVET	
121SC6	2012	*Citrobacter freundii*	KPC-2	50	NO TC	urban effluent	VISAVET	
121SC62	2012	*Enterobacter asburiae*	KPC-2	ND	ND	urban effluent	VISAVET	
121SC65	2012	*Klebsiella pneumoniae*	KPC-2	70	NO TC	urban effluent	VISAVET	1
121SC66	2012	*Klebsiella oxytoca*	KPC-2	ND	NO TC	urban effluent	VISAVET	1
121SC68	2012	*Kluyvera cryocrescens*	KPC-2	50	NO TC	urban effluent	VISAVET	1

121SC7	2012	*Klebsiella oxytoca*	KPC-2	ND	ND	urban effluent	VISAVET	1
121SC72	2012	*Klebsiella oxytoca*	KPC-2	ND	NO TC	urban effluent	VISAVET	1
121SC73	2012	*Citrobacter freundii*	KPC-2	70	NO TC	urban effluent	VISAVET	1
121SC80	2012	*Citrobacter freundii*	KPC-2	ND	ND	urban effluent	VISAVET	
121SC81	2012	*Raoultella ornithinolythica*	KPC-2	ND	ND	urban effluent	VISAVET	
121SC84	2012	*Raoultella ornithinolythica*	KPC-2	ND	NO TC	urban effluent	VISAVET	
121SC85	2012	*Klebsiella oxytoca*	KPC-2	ND	ND	urban effluent	VISAVET	1
131SC19	2013	*Enterobacter asburiae*	KPC-2	ND	ND	urban effluent	VISAVET	1
131SC2	2013	*Raoultella ornithinolytica*	KPC-2	ND	ND	urban effluent	VISAVET	1
131SC23	2013	*Citrobacter freundii*	KPC-2	ND	ND	urban effluent	VISAVET	
131SC24	2013	*Klebsiella oxytoca*	KPC-2	ND	ND	urban effluent	VISAVET	1
131SC31	2013	*Citrobacter freundii*	KPC-2	ND	ND	urban effluent	VISAVET	1
131SC4	2013	*Klebsiella oxytoca*	KPC-2	ND	ND	urban effluent	VISAVET	1
131SC42	2013	*Citrobacter freundii*	KPC-2	ND	ND	urban effluent	VISAVET	
131SC48	2013	*Enterobacter asburiae*	KPC-2	ND	ND	urban effluent	VISAVET	1
131SC8	2013	*Kluyvera ascorbata*	KPC-2	ND	ND	urban effluent	VISAVET	1
37C1	2011	*Citrobacter freundii*	KPC-2	40	NO TC	urban effluent	VISAVET	
51CX1	2011	*Citrobacter freundii*	KPC-2	40	NO TC	urban effluent	VISAVET	
72CX1	2011	*Citrobacter freundii*	KPC-2	40	NO TC	urban effluent	VISAVET	
74CX1	2011	*Citrobacter freundii*	KPC-2	40	NO TC	urban effluent	VISAVET	1
88cz1	2011	*Kluyvera cryocrescens*	KPC-2	40/300	NO TC	urban effluent	VISAVET	1
93CX1	2011	*Enterobacter cloacae*	KPC-2	40	NO TC	urban effluent	VISAVET	1
1001697	2010	*Citrobacter freundii*	KPC-2	50	NO TC	clinical isolate	H. La Paz	1
09064836	2009	*Citrobacter freundii*	KPC-2	50	NO TC	clinical isolate	H. La Paz	1
10000126	2010	*Citrobacter freundii*	KPC-2	50	NO TC	clinical isolate	H. La Paz	1

*, conjugation experiments were only performed for the isolates from 2011 and 2012 using recipient strain, *E. coli* BM21 (Nalidixic and Rifampin resistant). NO TC indicates that the conjugation experiment failed and ND not determined, Even in the cases of conjugation failure, the hybridization with an specific probe for blaKPC-2 was done and the result is indicated in the column S1 nuclease.

Supplementary Table 2: Sequencing results

Isolate	Microorganism	Year	Source	GC content (%)	N50	Contigs	Total of contigs	Read_length	Reads count	Total matched bases	Average Coverage	Sample accession	Secondary accession	Run accession
9064836	*Citrobacter freundii*	2009	clinical	51.9	147184	162	5457494	183.34	4830238	639680647	117	ERS1696190	SAMEA104032297	ERR1950605
10000126	*Citrobacter freundii*	2010	clinical	51.9	168550	121	5422873	177.45	8598616	1113253923	205	ERS1696191	SAMEA104032298	ERR1950606
10016917	*Citrobacter freundii*	2010	clinical	51.8	181586	126	5396670	182.10	5796626	822604837	152	ERS1696192	SAMEA104032299	ERR1950607
74CX1	*Citrobacter freundii*	2011	urban effluent	51.9	128562	124	5122138	183.41	5806124	841826099	164	ERS1696189	SAMEA104032296	ERR1950604
121SC21	*Citrobacter freundii*	2012	urban effluent	51.6	81928	173	5520208	175.66	5523492	835732399	151	ERS1696268	SAMEA104032375	ERR1950683
121SC54	*Citrobacter freundii*	2012	urban effluent	51.8	74540	220	5163958	152.89	11566822	1151473983	223	ERS1696272	SAMEA104032379	ERR1950687
121SC73	*Citrobacter freundii*	2012	urban effluent	51.6	180850	113	5401577	173.47	8381070	1129536386	209	ERS1696278	SAMEA104032385	ERR1950693
131SC31	*Citrobacter freundii*	2013	urban effluent	51.8	98982	135	5000525	168.35	8903466	1256276522	251	ERS1696284	SAMEA104032391	ERR1950699
121SC57	*Citrobacter braakii*	2012	urban effluent	52.1	110562	151	5283838	193.10	3651470	685351526	130	ERS1696273	SAMEA104032380	ERR1950688
121SC13	*Citrobacter farmeri*	2012	urban effluent	52.8	169462	132	5444896	185.25	4781478	858801658	158	ERS1696267	SAMEA104032374	ERR1950682
121SC3	*Enterobacter asburiae*	2012	urban effluent	54.4	102335	154	5275901	181,00	3598944	626166160	119	ERS1696269	SAMEA104032376	ERR1950684
121SC32	*Enterobacter asburiae*	2012	urban effluent	55.8	95772	144	4900317	185.03	2969666	533591321	109	ERS1696270	SAMEA104032377	ERR1950685
131SC19	*Enterobacter asburiae*	2013	urban effluent	55.6	93831	175	4782468	183.40	3493378	617498154	129	ERS1696281	SAMEA104032388	ERR1950696
131SC48	*Enterobacter asburiae*	2013	urban effluent	55.4	74566	175	5045048	181.07	3472124	606447632	120	ERS1696285	SAMEA104032392	ERR1950700
93CX1	*Enterobacter cloacae*	2011	urban effluent	54.7	78763	171	5112135	174.78	4616324	766108155	150	ERS1696288	SAMEA104032395	ERR1950703
121SC07	*Klebsiella oxytoca*	2012	urban effluent	55.5	75531	226	6080824	179.43	6771958	964297833	159	ERS1696265	SAMEA104032372	ERR1950680
121SC66	*Klebsiella oxytoca*	2012	urban effluent	55.8	93845	237	6644824	177.18	7239422	1031939813	155	ERS1696275	SAMEA104032382	ERR1950690
121SC72	*Klebsiella oxytoca*	2012	urban effluent	55.7	101687	165	6494558	172.99	9024738	1372538926	211	ERS1696277	SAMEA104032384	ERR1950692
121SC85	*Klebsiella oxytoca*	2012	urban effluent	55.6	100855	201	6210499	163.04	8697124	1229782113	198	ERS1696279	SAMEA104032386	ERR1950694
131SC04	*Klebsiella oxytoca*	2013	urban effluent	55.5	93868	189	6192754	195.20	2474682	390697967	63	ERS1696280	SAMEA104032387	ERR1950695
131SC24	*Klebsiella oxytoca*	2013	urban effluent	55.7	101669	157	6270776	155.14	7985718	1098494031	175	ERS1696283	SAMEA104032390	ERR1950698
121SC65	*Klebsiella pneumoniae*	2012	urban effluent	57.0	62666	242	5568593	180.85	3971586	631596771	113	ERS1696274	SAMEA104032381	ERR1950689
131SC8	*Kluyvera ascorbata*	2013	urban effluent	53.6	79930	181	5511169	173.88	3833918	632886374	115	ERS1696286	SAMEA104032393	ERR1950701
88CZ1	*Kluyvera cryocrescens*	2011	urban effluent	53.6	187948	97	5255144	177.67	4905944	826624124	157	ERS1696287	SAMEA104032394	ERR1950702
121SC68	*Kluyvera cryocrescens*	2012	urban effluent	53.6	105247	153	5209659	167.52	3706848	587882933	113	ERS1696276	SAMEA104032383	ERR1950691
121SC10	*Raoultella ornithinolythica*	2012	urban effluent	54.7	107811	144	6502063	183.05	6542840	1007807096	155	ERS1696266	SAMEA104032373	ERR1950681
121SC39	*Raoultella ornithinolythica*	2012	urban effluent	55.0	130169	126	6079398	182.08	4614396	805517527	132	ERS1696271	SAMEA104032378	ERR1950686
131SC2	*Raoultella ornithinolythica*	2013	urban effluent	55.3	96097	195	6149508	180.23	3727140	640163211	104	ERS1696282	SAMEA104032389	ERR1950697

Supplementary Table 3: Plasmids and Resistance genes

Isolate	Microorganism	Year	Plasmid size based on s1-hybridization (kb) *	Genome *in silico* MLST	Sequence of *bla* KPC-2	Plasmids				Antibiotic Resistance Genes																					
						KPC-2 plasmid type IncP(6)	KPC-2 plasmid other Inc Types	Plasmid-1919 bp	Additional plasmids	*aac(6')Ib-cr*	*aacA4*	*aadA1*	*aadA2*	*aadA7*	*aph(3')-Ia*	*blaKPC-2*	*ΔblaTEM-1A*	*blaOXA-10*	*blaBEL-1*	*blaCTX-M*	*blaFOX-*	*blaOXY-*	*other beta-lactamases*	*catB2*	*dfrA*	*fosA*	*mph(A)*	*Qnr*	*strB*	*sul1*	*other genes*
9064836	*Citrobacter freundii*	2009	50	Unknown ST	+	+		+	IncFI, IncFII	✓	✓		✓	✓	✓	✓		✓		*CTX-M-53*	*blaFox-4*		*blaCMY-71*		*dfrA12*		✓	*QnrB43*	✓	✓	*aac(3)-IIa, aac(6')-31*
10016917	*Citrobacter freundii*	2010	50	Unknown ST	+	+		+	IncFI, IncFII					✓	✓	✓	✓						*blaCMY-71*				✓	*QnrB43*	✓	✓	*aac(3)-IIa, aac(6')-31*
10000126	*Citrobacter freundii*	2010	50	Unknown ST	-			+	IncFI, IncFII, Col (Ye4449)	✓	✓		✓	✓	✓		✓			*CTX-M-53*			*blaCMY-71*		*dfrA12*		✓	*QnrB43*	✓	✓	*aac(3)-IIa, aac(6')-31*
74CX1	*Citrobacter freundii*	2011	40	Unknown ST	+	+										✓	✓						*blaCMY-2*					*QnrB32*			
121SC21	*Citrobacter freundii*	2012	50	Unknown ST	+	+			IncN [ST-9]	✓						✓	✓	*blaOXA-1*					*blaCMY-41*	*catB3*			✓			✓	*ARR-3*
121SC54	*Citrobacter freundii*	2012	50 *	Unknown ST	+		IncN		IncFI, IncQ2	✓	✓					✓	✓	✓			*blaFOX-3*		*blaCMY-41*								
121SC73	*Citrobacter freundii*	2012	70	Unknown ST	+		IncU		Col(MGD2)	✓	✓					✓					*blaFOX-7*		*blaCMY-73*					*QnrB49*		✓	
131SC31	*Citrobacter freundii*	2013	ND	Unknown ST	+	+		+	IncFII, IncN2, Col (Ye4449)	✓	✓	✓			✓	✓	✓						*blaCMY-79*		*dfrA1*		✓			✓	
121SC57	*Citrobacter braakii*	2012	ND	Unknown ST	+	+			IncFI, IncN, IncR	✓	✓					✓	✓	✓	✓				*blaCMY-70*					*QnrB40*			
121SC13	*Citrobacter farmeri*	2012	ND	NA	+	+			IncN2							✓	✓						*blaIMP-8*				✓	*QnrS2*		✓	
121SC3	*Enterobacter asburiae*	2012	ND	Unknown ST	+	+			IncFI, IncHI2	✓	✓				✓	✓										✓		*QnrA1*		✓	*aadB*
121SC32	*Enterobacter asburiae #*	2012	50	*E. cloacae* ST-23	+	+		+	ColRNAI	✓	✓					✓	✓	✓					*blaACT-1*			✓	✓			✓	*dfrB3*
131SC19	*Enterobacter asburiae #*	2013	ND	*E. cloacae* ST-23	+	+			IncN2	✓	✓					✓	✓	*blaOXA-2*					*blaACT-1*			✓				✓	
131SC48	*Enterobacter asburiae #*	2013	ND	*E. cloacae* ST-23	+	+				✓	✓					✓	✓	*blaOXA-2*					*blaACT-1*			✓				✓	
93CX1	*Enterobacter cloacae*	2011	40	Unknown ST	+	+			IncX3, repA (pKPC-2)			✓				✓	✓									✓					
121SC07	*Klebsiella oxytoca*	2012	ND	ST-77	+	+			IncFI, IncFII, IncQ2, IncU, Col(Ye4449)	✓	✓					✓	✓	✓			*blaFOX-3*	*OXY-5-2*									
121SC66	*Klebsiella oxytoca*	2012	ND	ST-50	-				IncFI, IncFII, IncU				✓		✓				✓			*OXY-1-2*	*blaGES-5*							✓	
121SC72	*Klebsiella oxytoca*	2012	ND	Unknown ST	+	+		+	IncL/M, Col(IRGK)	✓	✓		✓		*aph(3')-Ic*	✓	✓					*OXY-1-5*								✓	*aac(3)-I, cmlA1*
121SC85	*Klebsiella oxytoca*	2012	ND	Unknown ST	+	+		+	IncFI, IncFII, ColE10	✓	✓					✓	✓	✓	✓										✓		*aac(3)-Ib, strA*
131SC04	*Klebsiella oxytoca*	2013	ND	Unknown ST	+		IncN [ST-6]		Col(MGD2)							✓						*OXY-5-1*			*dfrA14*			*QnrS1*			
131SC24	*Klebsiella oxytoca*	2013	ND	ST-135	+		IncX3		IncFI, Col(MGD2)			✓			✓	✓	✓					*OXY-1-3*									
121SC65	*Klebsiella pneumoniae*	2012	70	ST-1200	+		IncN [ST-7]		InFI, IncFII, IncR, Col(Ye4449)							✓							*blaSHV-82*		*dfrA14*	✓		*QnrS1*			*oqxA, oqxB*
131SC8	*Kluyvera ascorbata*	2013	ND	NA	+	+			IncFI, IncHI1, Col (MGD2)	✓		✓				✓	✓	*blaOXA-9*		*CTX-M-5*								*QnrS1*			*aac(6´)-Ib*
88CZ1	*Kluyvera cryocrescens*	2011	40/300	NA	+	+										✓	✓														
121SC68	*Kluyvera cryocrescens*	2012	50	NA	+	+			Col(IRGK)							✓	✓														
121SC10	*Raoultella ornithinolythica*	2012	50 *	NA	+	+		+	IncHI1 [ST-11], IncN[ST-7], Col(MGD2)	✓	✓	✓			*aph(3')-XV*	✓	✓						*blaVIM-1*	✓							
121SC39	*Raoultella ornithinolythica*	2012	50 *	NA	+	+			IncFIA, IncN[ST-7]	✓	✓	✓			*aph(3')-XV*	✓	✓						*blaVIM-1*	✓	*dfrA14*	✓	✓	*QnrS1*		✓	
131SC2	*Raoultella ornithinolythica*	2013	ND	NA	+	+			IncFIA, IncA/C, ColRNAI	✓	✓	✓				✓	✓		✓					✓		✓				✓	

*, transconjugants were obtained and contained a 50 Kb plasmid; #, belogned to *E. cloacae* beased on genome analysis.
